# Factors Affecting the Germination Ecology of *Pimelea trichostachya* and Its Relationship to Field Emergence

**DOI:** 10.3390/plants12112112

**Published:** 2023-05-26

**Authors:** Rashid Saleem, Shane Campbell, Mary T. Fletcher, Sundaravelpandian Kalaipandian, Steve Adkins

**Affiliations:** 1 School of Agriculture and Food Sciences, University of Queensland, Gatton, QLD 4343, Australia; 2 Queensland Alliance for Agriculture Innovation, University of Queensland, Coopers Plains, QLD 4108, Australia

**Keywords:** *Pimelea trichostachya*, dormancy, germination, GA_3_, light, temperature, osmotic stress, seed burial depth

## Abstract

*Pimelea trichostachya* Lindl is a little-understood Australian native plant, with irregular field emergence, causing significant poisoning to grazing livestock. The study aims to examine the form of dormancy exhibited by *P. trichostachya* and determine how key environmental conditions, such as alternating temperature and light conditions, moisture availability, substrate pH and burial depth, affect its germination and emergence. The study concludes that *P. trichostachya* has a complex dormancy mechanism. This comprises a physical component that can be partly removed by fruit scarification, a metabolic dormancy that can be overcome by gibberellic acid (GA_3_), and a suspected third mechanism based on a water-soluble germination inhibitor. The results showed that scarified single seeded fruit (hereafter seed) with GA_3_ treatment gave the highest germination percentage (86 ± 3%) at 25/15 °C, with good germination rates at other temperature regimes. Light exposure stimulated germination, but a significant proportion of seeds still germinated in the dark. The study also found that seeds could germinate under water-limited conditions and a wide range of pH levels (4 to 8). Seedling emergence was inhibited when seeds were buried below 3 cm in soil. *Pimelea trichostachya* emergence in the field commonly occurs from Autumn to Spring. Understanding its dormancy mechanism and recognizing its triggers for germination will enable better prediction of outbreaks. This can help landholders prepare for emergence and help manage seedbank build-up in pastures and crops.

## 1. Introduction

The genus *Pimelea* consists mainly of small shrub or forb species that are commonly known as rice flowers. This genus is indigenous to Australia, Lord Howe Island, New Zealand, and the island of Timor. From herbarium records and various botanical studies, 90 *Pimelea* species are present across New South Wales [1], Queensland (QLD) [2], South Australia [3], Northern Territory [4], and Western Australia [5]. Four have agronomic importance [*viz. P. simplex* F. Muell. subspecies *simplex*, *P. simplex* subspecies *continua* (J.M. Black) Threlfall, *P. trichostachya* (Lindl.) and *P. elongata* (Threlfell)], as they contain the toxin simplexin, a diterpenoid orthoester that is toxic to cattle [6,7]. Historically known as Marree Disease or St. George Disease, it has been a prevailing problem throughout the arid grazing regions of inland Australia [8]. Cattle grazing pastures where *Pimelea* is present are affected by pulmonary venule constriction and potentially fatal heart impacts with fluid leakage causing the characteristic subcutaneous oedema associated with *Pimelea* poisoning. All livestock consuming this plant also experience severe diarrhea [7,8]. 

The distribution of *P. trichostachya* is broader compared to the other toxic species [5]. Consequently, this species is generally considered the most problematic species. *Pimelea trichostachya* typically grows between 20 and 60 cm tall and produces large amounts of self-pollinated seeds [9]. Generally, it grows well in dense buffel grass [*Pennisetum ciliare* (L.) Link] pastures and prefers sandy and acidic soils [7]. However, its appearance in the field is often sporadic with no known trigger for its germination. It is known that *P. trichostachya* possesses a complex but uncharacterized dormancy mechanism that presumably plays a role in its seedbank persistence and its sporadic field appearance. Little is known concerning the environmental requirements that trigger its germination and emergence in the field. Exposure to natural weathering conditions has been found to enhance the germinability of its single seeded fruit (hereafter referred to as seeds) for all three species. *Pimelea trichostachya* seeds, which likely possessed a strong physical dormancy, took more than 3 months to germinate. *Pimelea simplex* seeds exhibited an even deeper dormancy, as they did not germinate even after 9 months of weathering. Similarly, laboratory-stored seeds of this species did not germinate for at least 18 months after storage [7]. On the other hand, *P. elongata* seeds, presumably having a weaker physical dormancy, required less than 2 months of weathering to initiate germination.

Although some information about the germination requirements of *P. trichostachya* is available from earlier studies [10], little is available on the effects of alternating temperature, light, or various soil moisture or pH conditions. From experiments conducted by Silcock and Mann [10], it is known that seeds of *P. trichostachya* possess dormancy, and can germinate best at 25/15 °C. In that study, chemical stimulation of germination was achieved using gibberellic acid (GA_3_) and smoke water, and physical treatments to break dormancy included scarification, fruit coat removal, cold stratification, and heat to attain a sizeable level of germination. Further studies revealed that scarification or dry heat or cold stratification alone could not overcome dormancy. Additionally, Silcock [11] reported that prolonged moist conditions and topsoil disturbance appear to be the common triggers of successful germination in the field. Another study reported that GA_3_ could enhance germination of *P. trichostachya* seeds, but only when the fruit coats had been weakened by nicking [12]. 

As *Pimelea* species are native to Australia, there is currently no legislation in place to control or contain their growth. However, implementing preventive measures and practicing good farm biosecurity procedures can play a vital role in managing these poisonous plants. *Pimelea* seeds are dispersed by wind, so it is essential to physically hand-pull the plants before they flower or form seed. Hand-pulling may not be a practical option for large-scale control, but it can still be an effective method for managing *Pimelea* plants in a small paddock. Proper disposal of the pulled plants is crucial to prevent their re-growth and the production of seed. The alternative methods, such as mechanical removal, herbicides, or controlled grazing can be opted for depending on the specific circumstances and available resources. The most effective approach to controlling *Pimelea* is to prevent its germination during the Autumn and Winter months by avoiding the conditions that trigger its germination. It is worth noting that the use of contaminated pasture seeds has been associated with the introduction of *Pimelea* in previously uncontaminated paddocks [7]. For example, seeds of *P. trichostachya* can mix with buffel grass seeds, leading to ongoing infestations of this problematic species. Therefore, when establishing new pastures, it is essential to exercise caution and source clean, *Pimelea*-free pasture seeds.

The objectives of the study are to further understand seed dormancy, germination ecology, and seedling emergence characteristics of *P. trichostachya* to develop a suitable management approach for predicting when it will emerge from the seedbank. The study aims to examine the form of dormancy exhibited by *P. trichostachya* and determine how key environmental conditions, such as alternating temperature and light conditions, moisture availability, substrate pH, and burial depth affect its germination and emergence. To achieve the first objective of understanding dormancy, the study involved conducting germination tests using different treatments, such as scarification and chemical treatments, to determine the type of dormancy exhibited by the seeds. This information can help identify the optimal conditions for breaking dormancy and improving germination rates. The second objective of understanding how environmental conditions affect germination and emergence involved conducting experiments with different treatments, such as varying temperature and light conditions, moisture availability, substrate pH, and burial depth. By observing the response of *P. trichostachya* to these treatments, researchers can identify the optimal conditions for promoting germination and emergence.

Overall, by achieving these objectives, researchers can develop a better understanding of the germination ecology of *P. trichostachya* and formulate management strategies that can effectively control the growth of the plant in agricultural and natural environments.

## 2. Results

### 2.1. Microscopic Observations

**Figure 1:**
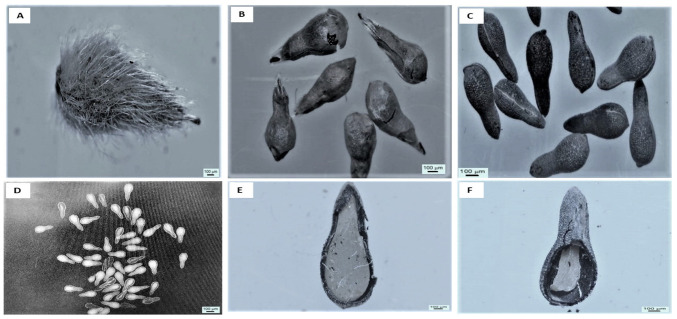
Microscopic images of *P. trichostachya*; (**A**) intact single seeded fruit, (**B**) fruit after the removal of the thin hairy exocarp but showing the paper-like mesocarp layer (achieved by sandpaper scarification), (**C**) seed encased by the hard endocarp and (**D**) an X-ray image of these scarified seed showing 70% seed fill, and (**E**) a fully ripened embryo surrounded by the hard endocarp and a paper-thin testa and (**F**) an underdeveloped embryo.

The microscopic images of *P. trichostachya* seeds provide a detailed visual representation, offering insights into the seeds’ internal structures (Figure 1). Image (A) displays the intact seed, giving an initial overview of its composition. Image (B) reveals the seed after the removal of the thin, hairy exocarp, exposing the underlying paper-like mesocarp achieved through sandpaper scarification. In image (C), the seed is observed enclosed within a protective hard endocarp, ensuring its safety. The X-ray image (D) shows scarified seeds with approximately 70% filling of the seeds. Image (E) captures a fully ripened embryo surrounded by a resilient hard endocarp and a delicate, paper-thin testa, showcasing the intricacies of its protective structures. Lastly, image (F) presents an underdeveloped embryo, implying the need for further growth and maturation. Overall, these microscopic images provide valuable insights into the complex anatomy and developmental stages of *P. trichostachya* seeds, highlighting protective layers, seed fill percentage, and varying levels of embryo development. 

### 2.2. Effect of Various Dormancy Breaking Treatments

**Figure 2:**
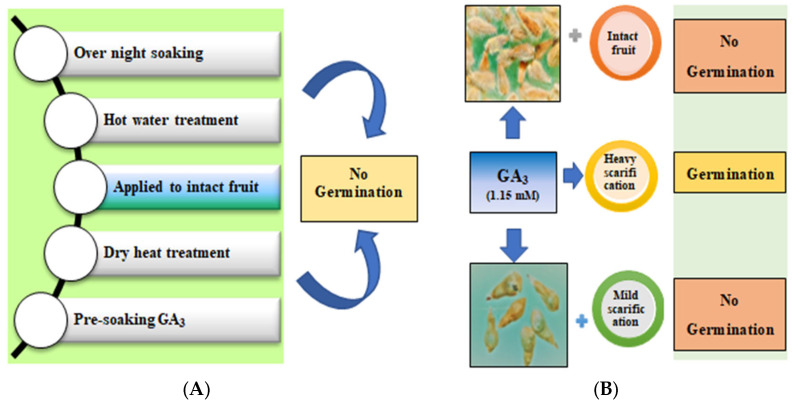
(**A**) The initial dormancy breaking methods attempted, and (**B**) those based on gibberellin (GA_3_) imbibition of intact and scarified seeds of *P. trichostachya* (after 56 days).

The data show *P. trichostachya* seed to possess a complex dormancy mechanism. Initial trials were conducted on intact seed and involved overnight soaking in tap water, a hot water treatment, a dry heat treatment, and a pre-soaking with GA_3_ to overcome dormancy, but no germination was recorded (Figure 2). However, a maximum germination percentage (86 ± 3%) could be achieved after a scarification treatment to weaken the hard endocarp (presumably overcoming a physical dormancy mechanism) and following a GA_3_ (1.15 mM) treatment (to overcome a physiological dormancy; Table 1).

**Figure 3:**
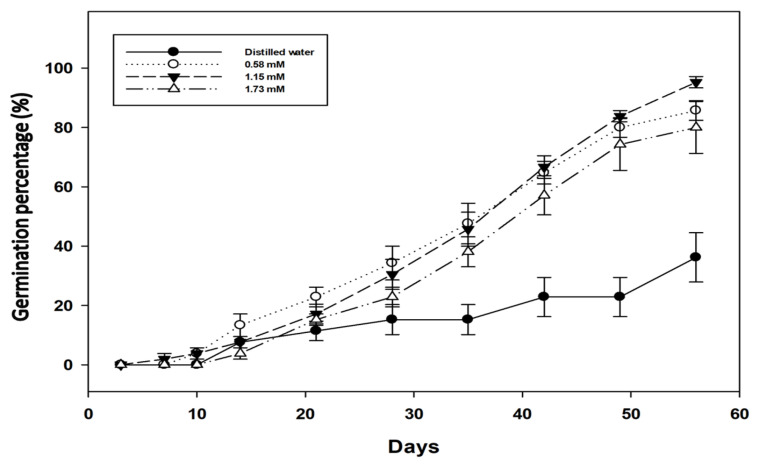
The cumulative germination of *P. trichostachya* (for 56 days) single seeded fruits imbibed in different concentrations of GA_3_ (0.00, 0.58, 1.15 and 1.73 mM). Results based on pooled data from two experiments.

An X-ray test showed that the remaining ungerminated portion of the seed (14%) was filled, presumably viable but still dormant. When applying this combined scarification and GA_3_ treatment, the germination rate was slow, remaining below 20% for the first 21 days of imbibition and only progressively increasing after this time (Figure 3), indicating the possibility of deeper than average physical or physiological dormancy, or a third dormancy mechanism, requiring imbibition time before it is overcome.

### 2.3. Effects of Temperature and Light on Germination 

**Figure 4:**
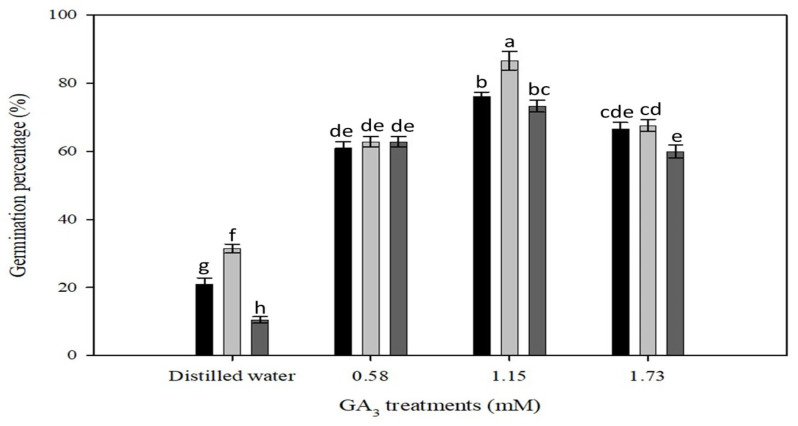
The combined effect of various temperature treatments (20/10 °C black bars, 25/15 °C light grey bars and 30/20 ± 2 °C dark grey bars) on the germination of *P. trichostachya*. The single seeded fruits were imbibed under a 12/12 h (day/night) photoperiod during the germination treatments. Data shown are from two experiments, each experiment consisting of three replicates of 25 fruits and bars indicate means ± SEM. Similar letters above bars indicate insignificance (*p* > 0.05).

The treatments showed a significant interactive effect of temperature and GA_3_ concentrations on the germination of *P. trichostachya* (*p* < 0.05; Figure 4). The germination of *P. trichostachya* at 25/15 °C was significantly higher than at 20/10 °C and 30/20 °C alternating temperatures. However, germination of *P. trichostachya* at 20/10 °C and 30/20 °C was statically on par with 25/15 °C. 

At a thermoperiod of 20/10 °C with a matching photoperiod of 12/12 h (day/night), a maximum germination percentage (76 ± 1%) was recorded when seeds of *P. trichostachya* were treated with 1.15 mM GA_3_, followed by those treated with 1.73 (66 ± 2%) or 0.58 mM GA_3_ (60 ± 2%). At 25/15 °C with a matching photoperiod of 12/12 h (day/night), the maximum germination percentage (86 ± 3%) was recorded when seeds were treated with 1.15 mM GA_3_, followed by those treated with 1.73 mM (67 ± 2%) or 0.58 mM GA_3_ treatment (62 ± 1%) (Figure 4). At 30/20 °C with a matching photoperiod of 12/12 h (day/night), the maximum germination percentage (73 ± 2%) was recorded when seeds were treated with 1.15 mM GA_3_, followed by those treated with 1.73 mM (59 ± 2%) or 0.58 mM (63 ± 1%) (Figure 4). 

**Figure 5:**
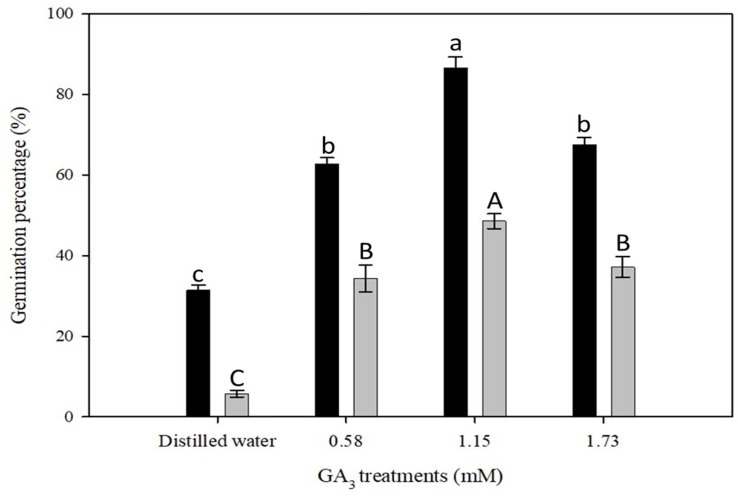
The combined effect of GA_3_ concentration and light on the germination of *P. trichostachya*. The single seeded fruit were imbibed under a thermoperiod of 25/15 °C with a matching 12/12 h (day/night) photoperiod (black bars) and under complete darkness (grey bars). Data were from two experiments, each consisting of three replicates of 25 fruits per replicate and bars indicate means ± SEM. Similar letters above bars indicate insignificance (*p* > 0.05).

There was a significant effect of light and GA_3_ concentrations on the germination of *P. trichostachya* (*p* < 0.05; Figure 5). A similar response to GA_3_ concentration was seen in darkness as in light (Figure 5), but at a *ca*. 50% lower rate. The highest germination was recorded for 1.15 mM GA_3_ treatments, followed by 1.73 mM and 0.58 mM GA_3_ treatments, which were not significantly different (*p* > 0.05) from each other under either alternating light or constant dark conditions (Figure 5).

### 2.4. Effect of Osmotic Stress on Germination

**Figure 6:**
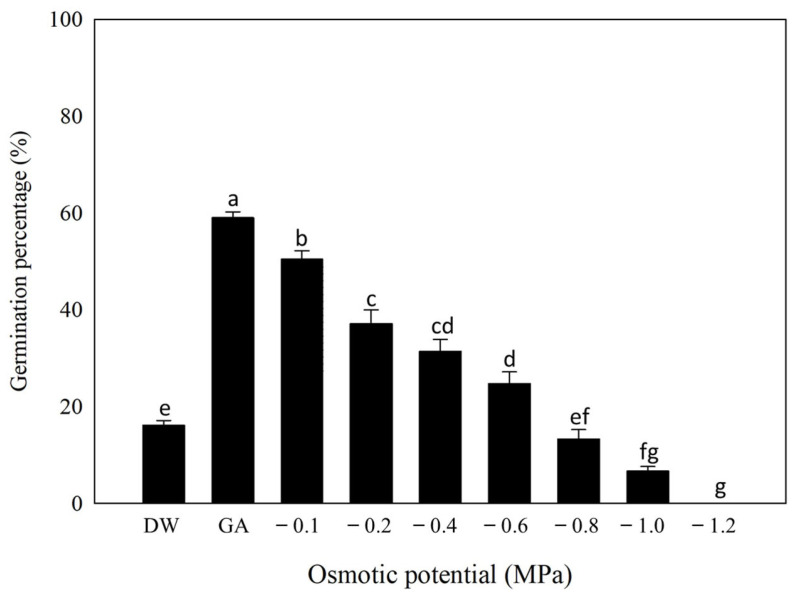
The combined effect of various osmotic potential treatments on the germination of *P. trichostachya*. The single seeded fruits were scarified and imbibed with GA_3_ (1.15 mM) under a thermoperiod of 25/15 °C with a matching 12/12 h photoperiod. Data shown are from two experiments, each consisting of three replicates of 25 fruits and bars indicate means ± SEM. Similar letters above bars indicate insignificance (*p* > 0.05).

There was a significant effect of osmotic stress on the germination of scarified and GA_3_-stimulated *P. trichostachya* seeds (Figure 6). The maximum germination was observed under the no water stress condition (60 ± 1%) and decreased as the osmotic potential of the germination environment decreased to −1.2 MP. Although some seed germinated at −1.0 MPa (50 ± 2%), germination was shown to be completely inhibited at −1.2 MPa.

The data suggested that an osmotic potential of *ca*. −0.6 MPa was required to inhibit 50% of the maximum germination as compared to the control (Figure 6). These results indicate that some *P. trichostachya* seeds are likely to germinate at relatively high osmotic stress (low soil moisture) conditions.

### 2.5. Effect of pH on Germination

**Figure 7:**
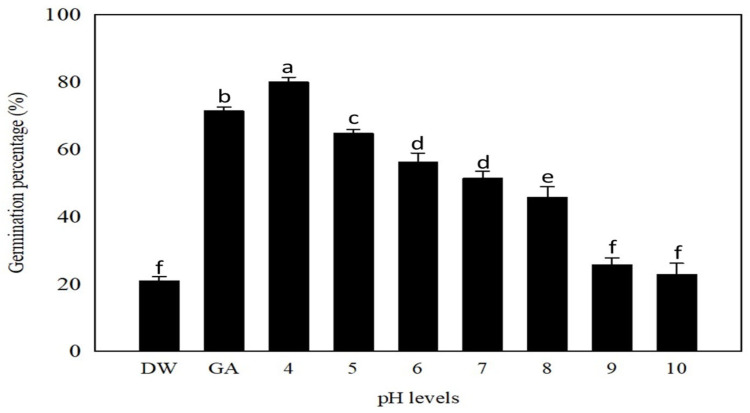
The effect of pH on the germination of *P. trichostachya*. Scarified single seeded fruits were imbibed with distilled water (DW), GA_3_ (GA; 1.15 mM) or in pH-adjusted solutions, under a thermoperiod of 25/15 °C with a matching 12/12 h (day/night) photoperiod. Data shown are from two experiments, each consisting of three replicates of 25 fruits and bars indicate means ± SEM. Similar letters above bars indicate insignificance (*p* > 0.05).

There was a significant effect of pH on the germination of GA_3_-stimulated *P. trichostachya* seeds (*p* < 0.05; Figure 7). The maximum germination percentage (*ca*. 80 ± 3%) was recorded at the acidic pH level of 4.0. Germination then progressively decreased with increasing pH, culminating in the lowest germination percentage (*ca*. 22 ± 3%) being recorded at pH levels of 9.0 and 10.0. 

### 2.6. Effect of Different Burial Depths on Seedling Emergence 

**Figure 8:**
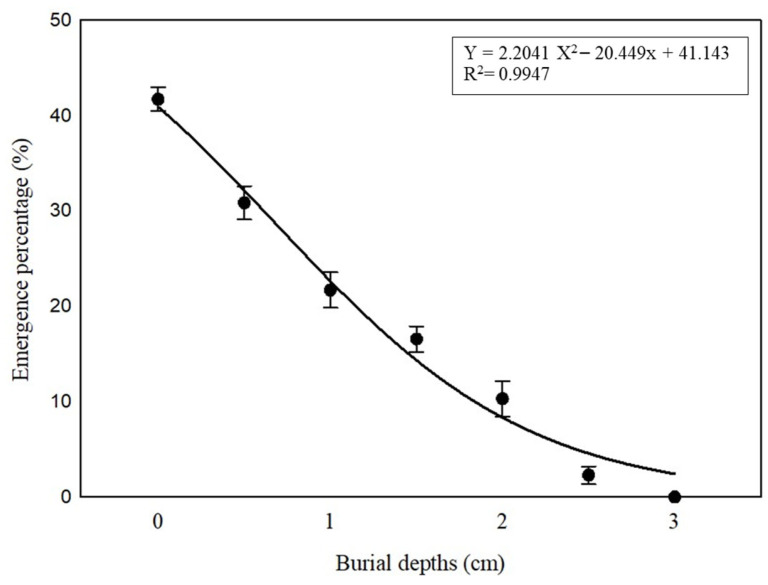
Effect of fruit burial depths on the cumulative emergence of *P. trichostachya* seedlings (for 56 days). The data is pooled from two experiments using scarified and GA_3_ (1.15 mM for 24 h) pre-soaked fruits. In each of the two experiments, there were 25 fruits per treatment and seven replications. Vertical bars indicate standard errors of the plotted means.

Analysis of variance revealed that there was a significant effect of burial depth on the emergence of *P. trichostachya* (*p* < 0.05; Figure 8). A strong negative relationship was observed between burial depth and seedling emergence (Figure 8). Maximum seedling formation (42 ± 1%) was recorded when the seed had been placed at the soil surface (0.0 cm), which then progressively decreased with the increase in burial depth, with no emergence being recorded from 3.0 cm depth (Figure 8). 

## 3. Discussion

This study suggests that the irregular field emergence observed in *P. trichostachya* populations is due to a complex dormancy mechanism (Figure 1A–F), which includes both physical and physiological components. This means that some seeds may not germinate immediately but instead remain dormant until certain conditions are met. This study also found that *P. trichostachya* seeds can germinate across a wide range of temperatures (Figure 4), which makes them suited for germination between Autumn and Spring in subtropical/tropical environments. However, the germination process can also be influenced by other environmental factors, such as light (Figure 5), soil moisture (Figure 6), pH (Figure 7), and seed burial depth (Figure 8). These findings highlight the importance of understanding the dormancy and germination requirements of *P. trichostachya* seeds, which can aid in developing effective management strategies. 

### 3.1. Dormancy Mechanisms

The results show *P. trichostachya* to have a complex dormancy mechanism, which would influence its time of emergence under various field conditions. The microscopic images offer valuable insights into the intricate anatomy and developmental stages of *P. trichostachya* seeds, shedding light on the protective layers, seed fill percentage, and varying levels of embryo development (Figure 1). These images provide a closer look at the protective layers surrounding the seed, such as the hairy exocarp and hard endocarp, which safeguard the seed during dormancy and unfavorable conditions. The images also reveal the seed fill percentage, giving us an understanding of the seed’s reproductive potential. Additionally, observing the varying levels of embryo development provides valuable information about the growth and maturation processes of *P. trichostachya*. These microscopic images are helpful in deepening our knowledge of the plant’s reproductive biology and its strategies for survival and germination in its environment.

Previous studies had reported *Pimelea* seeds to stay dormant in the soil for several years until favorable conditions occur to stimulate their germination [11]. In the present study, maximum germination (86 ± 3%) of *P. trichostachya* could only be achieved when both a scarification treatment, which weakened the hard seed endocarp, and a GA_3_ (1.15 mM) treatment were applied to overcome a physiological dormancy (Table 1, Figure 2). It is interesting to note that even with such treatments the germination was unable to reach 100% of the viable seeds and seeds were slow to germinate during the first 21 days of incubation and only progressively increased after this time in all treatments. Given this initial low germination but with increased germination after the first 21 days (Figure 3), a possible third form of dormancy is indicated, which is initially maintained by the presence of a germination inhibitor(s) in the seeds that needed to be lost from them before germination can proceed. Thus, the complex dormancy system present in *P. trichostachya* seeds would enable the formation of a persistent soil seedbank and would require certain climatic events or biological activities to break down the hard endocarp layer to modify aspects of metabolism and possibly periods of good rainfall to remove germination inhibitors from the seed. Exposure to different concentrations of GA_3_, applied to scarified seed, greatly improved germination (Figure 2). In the field, biotic and abiotic factors will act to overcome these dormancy mechanisms. As such, this may take different lengths of time depending upon the prevailing climate and dormancy mechanism. It will occur at different rates, with different cohorts of seed produced at the same site and in populations of plants growing in different sites. Thus, this staggered and unpredictable loss of dormancy could be an influential factor in causing the sporadic appearance of *P. trichostachya* seedlings in the field. However, other factors such as seed dispersal, environmental conditions, and competition with other plants could also play a role in the sporadic appearance of *P. trichostachya* seedlings in the field. Therefore, further research is warranted to fully understand the factors influencing the emergence of *P. trichostachya* seedlings in the field.

### 3.2. Temperature and Light

Once the dormancy is overcome, germination and seedling formation can occur under a certain range of conditions. Temperature and light are considered critical to initiate the germination process of some small-seeded species [13]. *Pimelea trichostachya* germinated well between alternating temperature regimes from 10/20 to 30/20 °C, peaking at 25/15 °C under illuminated conditions (Figure 4). Germination could take place in darkness after seeds have their dormancy removed (Figure 5). However, germination is enhanced under light, as compared to dark conditions (Figure 5). Two-fold higher germination was achieved in light indicating that *P. trichostachya* is positively photoblastic and well-adapted to germinating when seeds are located on or near the soil surface. This may be an advantageous adaptation in areas with variable or patchy vegetation as it allows the species to take advantage of gaps in the vegetation and quickly establish itself in the available space. The finding that seeds can also germinate in the dark has important implications for management strategies aimed at reducing the emergence and spread of *P. trichostachya* such as burying or covering seeds, which may not always be an effective means of control. In addition, it suggests that the species may have a degree of resilience to disturbances such as tillage or soil disturbance as buried seeds may still be able to germinate and establish in the future. Bajwa [14] reported that germination of parthenium weed (*Parthenium hysterophorus* L.) can take place in darkness after seeds overcome dormancy; however, the dormancy-breaking process is accelerated in the presence of light as compared to dark. The current study also concurs with the finding of Pereira et al. [15], who found that the effect of light and temperature on seed germination was pronounced with the application of plant growth regulators. Light and growth-promoting compounds (e.g., nitrate and GA_3_) can trigger the germination of seeds with physiological dormancy [16,17]. Overall, these findings suggest that *P. trichostachya* is well-adapted to the environmental conditions commonly found in QLD during Autumn and Spring months, which may provide optimal conditions for the species to germinate and establish, provided that favorable rainfall conditions occur during this period.

### 3.3. Osmotic Stress 

Osmotic stress is known to impact the germination of most species [18,19]. The present study also showed osmotic stress to be a key factor in determining the final germination percentage of non-dormant *P. trichostachya* seeds. The maximum germination response of *P. trichostachya* seeds was in the control condition with scarification, GA_3_ and with no water stress (Figure 6). The germination decreased as the osmotic potential of the germination environment increased. The data suggested that an osmotic potential of *ca*. −0.6 MPa was sufficient to inhibit 50% of its maximum germination. This value is higher than that reported for many weed species [20] suggesting that this native species may be more tolerant to water stress conditions than the various weed species present in the same pasture. It has been argued that species with the ability to germinate under water-limiting conditions will have a higher chance of growth and development in dry conditions as compared to neighboring species [21]. Thus, the ability of *P. trichostachya* to germinate under moderate water stress conditions could give it a competitive advantage under the typically dry conditions of the western QLD beef pastures. This is because *P. trichostachya* has the capacity to emerge earlier than many weed species present in the pasture, which may be less able to germinate and become established under conditions of moderate water stress. Therefore, the ecological context and specific conditions of each pasture should be carefully considered when assessing the potential impacts of *P. trichostachya* on the ecosystem.

### 3.4. Substrate pH

In terms of pH, the results of this study suggest that *P. trichostachya* can germinate under a wide range of soil pH conditions (i.e., pH 4.0 to 8.0), which is consistent with the pH of the soils where it is found growing [22]. Maximum germination (*ca.* 80 ± 5%) was recorded at the acidic pH level of 4.0 (Figure 7). Although germination declined as the pH increased from 5.0 (64 ± 1%) to 8.0 (45 ± 3%), the relatively high germination achieved over this range of normal soil conditions indicates that pH is not a limiting factor for *P. trichostachya* seed germination in most areas of Australia. *Pimelea trichostachya* is found in a variety of habitats, including sandy soils of varying color and acidity, as well as hard-setting duplex soils with a red, brown, or grey hue. These types of soils are commonly found in poplar/bimble box (*Eucalyptus populnea* F.Muell.) country [7]. This may be because of soil acidity on the structure or chemical composition of the seed coat, which could impact the ability of water and oxygen to penetrate the seed and initiate germination. However, it is also possible that other factors, such as changes in soil microbial communities or nutrient availability, may be contributing to the observed increase in germination under acidic conditions. Further research would be needed to fully understand the mechanisms behind this observation. 

### 3.5. Burial Depth

The emergence of seedlings from non-dormant *P. trichostachya* seeds was negatively impacted by increasing burial depths. In fact, no seedlings were produced when the seeds were buried at a depth of 3 cm (Figure 8). In other species, it has been shown that higher mechanical resistance provided by deep soil layers can impede early radicle growth and seedling elongation and subsequently decrease emergence [23]. In some situations (e.g., cropping), a management system utilizing a soil burial approach could be useful to place *P. trichostachya* seeds at a 3 cm depth or below in the soil profile and therefore reduce seedling emergence. In the longer term, such burial should lead to a reduction in the soil seedbank reserves [24]. Thus, to reduce the seedling appearance of this species in cropping, one strategy might be to undertake a strategic cultivation or use a residue management practice that helps bury seeds to a greater depth and in so doing limit seed exposure to light, which is an environmental trigger discovered to stimulate germination (Figure 8). However, it is important to consider the long-term effectiveness of burying seeds deeper in the soil as buried seeds may remain viable for many years and may still emerge as seedlings in subsequent growing seasons.

## 4. Materials and Methods

### 4.1. Seed Collection, Processing and Storage

**Figure 9:**
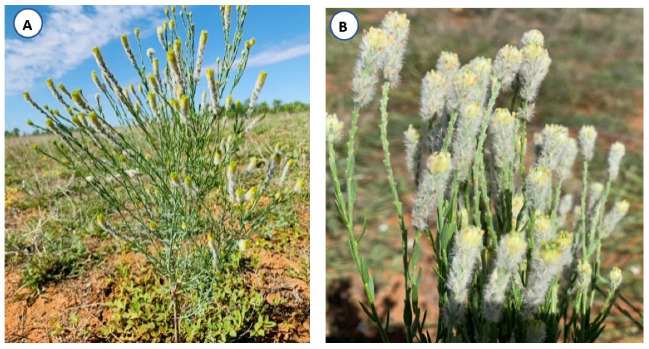
(**A**) A single *P. trichostachya* plant and (**B**) collection of *P. trichostachya* inflorescences growing on sandy soil near Roma, Queensland (images taken by Rashid Saleem).

Mature plants of *P. trichostachya*, bearing mature and immature fruits, were collected in September 2018 from a paddock that had been planted to forage oats (*Avena sativa* L.) in the Maranoa Shire located 76 km south of Roma (−27.22551167° S 148.5463033° E) in QLD. These plants were multi-branched, about 30 to 40 cm high, and were growing on red sandy soil. A voucher specimen (Figure 9A,B) was lodged with the QLD Herbarium for identification and was later confirmed to be *P. trichostachya* (allotted the BRI number AQ1010277).

All collected plants were sun-dried for 2 weeks, and then the mature seeds (single seeded fruit) were gently removed by shaking the plants over a paper sheet. The seeds were again sun-dried for a further week, then placed into cotton cloth bags, and kept under laboratory conditions (22 ± 2 °C) to attain constant moisture content. After 1 week, the seeds were cleaned from all other plant parts by sieving and blowing using a small electric fan. The cleaned single-seeded seeds were placed into labeled paper bags, and then the bags were placed into airtight plastic containers and stored in the dark at 15 ± 2 °C and 15 ± 5% relative humidity (RH) in a seed storage room until further use.

### 4.2. Seed Anatomical Analysis and Fill

Fruits with a fluffy exocarp layer (Figure 1A) were gently rubbed with fine sandpaper (grade P 240) for 60 s (Figure 1B) to remove the exocarp hairs and when required for 10 s to scarify the inner endocarp (Figure 1C) to remove physical dormancy. Care was taken not to damage the true seed inside, but scarification for 60 s was sufficiently robust to weaken the endocarp (Figure 1D). 

Five randomly selected lots of 50 scarified seeds were X-rayed (Faxitron MX-20 Imaging system, Lincolnshire, IL, USA) at 18 Kv for 20 s, the images were captured using Bioptics software, and the average seed fill percentage was determined (Figure 1D; 70 ± 5%). In addition, further samples of seed were cut longitudinally with a razor blade, and microscopic (Nikon SMZ745T) images were captured of filled (Figure 1E) and unfilled seeds (Figure 1F). In all germination experiments, seed lots were surface sterilized in a sodium hypochlorite solution (2%; v/v) for 5 min followed by a double rinse in sterile, distilled water (DW), and then they were blotted dry on clean filter paper. All germination results presented have been corrected for seed fill (70 ± 5%).

### 4.3. General Seed Germination Test Protocols

All germination tests were performed using three replicate lots of 25 seeds, with seeds evenly placed across the surface of Petri dishes (90 × 15 mm), each lined with a double layer of Whatman No.1 filter paper, and all experiments were repeated over time [25]. Filter papers had been moistened with 5 mL of either distilled water or selected GA_3_ solutions (i.e., 0.58, 1.15 or 1.73 mM). Two drops of a fungicide (Pervicure; 1.5 mL L^−1^) were added to each solution to prevent fungal growth. Petri dishes were placed into a transparent Sistema Klip container (7.5 L, Coles Supermarket Ltd., Burwood East, VIC, Australia) lined with a moistened paper towel, and with an airtight lid in place, to reduce water evaporation from the Petri dishes. The Petri dishes were then placed into a germination incubator (TRIL-750 Illuminated Refrigerator Incubators, Thermoline, Wetherill Park, Australia), and germination was observed for 56 days with the addition of water to the filter papers if needed. To simulate dark conditions, replicate Petri dishes were wrapped in three layers of aluminum foil and only opened once for a germination count at the end of the study. In all germination studies, seeds were considered to have germinated when radicle emergence reached 2 mm and seedling formation was counted when the plumule and cotyledons emerged.

### 4.4. Effect of Various Dormancy Breaking Treatments 

The dormancy mechanism(s) of *P. trichostachya* were investigated. Seeds were gently rubbed for 60 s with fine sandpaper to remove the exocarp hairs. Then, in some treatments, seeds were either rubbed for a further 10 s to scarify the inner endocarp, or they remained lightly scarified. These seed lots were X-rayed to determine seed fill percentage. X-ray imaging was employed to analyze the internal structure of the seeds and determine the amount of space occupied by the embryonic material within each seed lot. After the X-ray examination, the seeds were subjected to surface sterilization using NaOCl. This sterilization process helps eliminate potential pathogens or contaminants present on the surface of the seeds. Sodium hypochlorite, commonly known as bleach, is effective in reducing microbial load and ensuring a clean and sterile seed surface. Germination tests were performed using three replicate lots of 25 seeds, with seeds evenly placed across the surface of the Petri dishes. The filter papers had been moistened with 5 mL of either distilled water or selected GA_3_ solutions (i.e., 0.58, 1.15, or 1.73 mM). Two drops of a fungicide (Pervicure; 1.5 mL L^−1^) were added to each solution to prevent fungal contamination. Incubation was undertaken at 25/15 ± 2 °C with a matching 12/12 h (day/night) photoperiod (cool white fluorescent *ca*. 100 µmol m^−2^ s^−1^). After a total of 56 days of incubation, a final count of germinated seeds was taken and any seeds that had not yet germinated were evaluated for seed fill using X-ray analysis.

### 4.5. Effects of Temperature, and Light on Germination 

The effects of temperature and light regime on germination of *P. trichostachya* seeds were investigated. Seeds were scarified for 60 s to overcome physical dormancy and imbibed in one of four GA_3_ concentrations (0.00, 0.58, 1.15 or 1.73 mM) to overcome physiological dormancy and incubated under three different thermoperiods (day/night; 20/10, 25/15, and 30/20 ± 2 °C) each with a matching 12/12 h (day/night) photoperiod (cool white fluorescent *ca*. 100 µmol m^−2^ s^−1^) or placed under complete darkness. To achieve different thermoperiods, three identical germination incubators (see previous description in Section 4.3) were used. 

### 4.6. Effect of Osmotic Stress on the Germination 

The effects of osmotic stress on the germination of *P. trichostachya* seeds were tested. Aqueous solutions providing osmotic potentials of 0.0, −0.1, −0.2, −0.4, −0.6, −0.8, −1.0 and −1.2 MPa were prepared by using polyethylene glycol 6000 (PEG 6000; Sigma-Aldrich, St. Louis, MO, USA) as described by Michael [26]. For these germination tests, scarified seeds were used and imbibed in 5.0 mL of an adjusted osmotic solution containing GA_3_ (1.15 mM). Two control treatments were set up with the seeds: one with sterile distilled water alone and one with GA_3_ (1.15 mM). This study was conducted at a 25/15 ± 2 °C day/night thermoperiod with a matching 12/12 h photoperiod. 

### 4.7. Effect of pH on the Germination 

The effects of pH on the germination of *P. trichostachya* seeds were tested. Buffer solutions made up to provide pH values between 4.0 to 10.0 were prepared according to the method of Chachalis and Reddy [27]. Briefly, the solutions were made using a 2 mM solution of MES [2-(N-morpholino) ethane-sulphonic acid] adjusted with either 0.1 M hydrochloric acid or sodium hydroxide (NaOH) to obtain solutions of pH 4.0, 5.0, and 6.0. In addition, a 2 mM solution of HEPES [N-(2-hydroxymethyl) piperazine–N′–(2-ethane-sulphonic acid)] was prepared and adjusted with 0.1 M NaOH to obtain the buffer solutions of pH 7.0 and 8.0. Buffer solutions of pH 9.0 and 10.0 were prepared using a solution of 2 mM tricine [N-Tris (hydroxymethyl) methyl-glycine] adjusted with 0.1 M NaOH. A one-to-one mixture of each buffer solution with a GA_3_ (2.30 mM) solution was prepared and added to each Petri dish. Unbuffered sterile distilled water (pH 6.4) and GA_3_ (1.15 mM) were used as control treatments. This study was conducted with scarified seeds at a 25/15 ± 2 °C day/night thermoperiod with a matching 12/12 h photoperiod. 

### 4.8. Effect of Different Burial Depths on the Emergence of P. trichostachya

The effect of seed burial depth on the emergence of *P. trichostachya* seedlings was assessed. Scarification and a 24 h pre-treatment soaking in GA_3_ (1.15 mM) were applied to the seeds prior to this experiment being undertaken to overcome physical and physiological dormancy. This experiment comprised seven burial depths (*viz.*; 0.0, 0.5, 1.0, 1.5, 2, 2.5 and 3.0 cm). The soil was collected from a *P. trichostachya*-affected paddock in the Maranoa Shire (27°15′32″ S 148°34′26″ E; 250 m elevation) in April 2019. Immediately upon arrival at UQ, the soil was passed through a 5 mm sieve to remove all bulk particles and debris and was then dried in a glasshouse for 10 days. Black plastic pots (10 cm diameter) were filled with 0.75 kg of this dry soil and moistened to field capacity with tap water. Twenty-five seeds were then placed equidistantly into different pots with seven replicate pots per treatment. Once sown onto the soil surface, additional soil was added to cover the seeds to the desired depth. The pots were then transferred to a greenhouse and watered on every alternate day by hand. Seedling emergence counts were undertaken every second day for 56 days with seedlings being removed once identified. Control pots were set up with no added seed and demonstrated that there were no germinable *Pimelea* seeds present in the Maranoa Shire soil at the time of experimentation.

### 4.9. Experimental Design, Germination Calculations and Statistical Analyses

For all germination trials (experiments), after 56 days the germination percentage of all treatments was calculated using Equation (1) [28]. The final germination percentage achieved was corrected by the percentage seed fill (70 ± 5% fill seeds of *P. trichostachya*) by using a modified version of the viability-adjusted germination using Equation (2) [29].

Germination Percentage (G%) = (Total number of germinated seeds/Total seed) × 100,(1)

Total number of seeds adjusted = G (%) × 100/Seed fill (%)(2)

All experiments were arranged in a completely randomized design giving equal importance to all the treatments. The significance of means of individual treatment factors and their interactions was estimated through ANOVA in all experiments using Statistix 8.1. Data from the repeated experiments were subjected to ANOVA, and as no significant time-by-treatment interactions were found (*p* > 0.05), data sets were pooled for analysis and presentation. Means were separated using Fisher’s protected LSD test at *p* = 0.05. Thus, the treatment means were presented in bar charts with ± SE of means using Sigma Plot v. 14.5.

## 5. Conclusions

This study investigated the dormancy and germination mechanisms of *P. trichostachya*, an Australian native plant, and its response to various environmental conditions. The results showed that the dispersal unit of *P. trichostachya* was a single-seeded fruit possessing a complex dormancy mechanism, comprising physical and metabolic components and a suspected third mechanism, possibly due to a water-soluble germination inhibitor. The plant can germinate and form seedlings under a range of environmental conditions with a preference for moderate temperatures, light, and mildly acidic soils. The field emergence of *P. trichostachya* is sporadic, occurring when a given seedbank seed load had lost dormancy and environmental conditions are suitable for germination, which generally occurs from Autumn to Spring in subtropical environments. These findings can help landholders anticipate seedling emergence and manage the build-up of *P. trichostachya* seedbanks, which can be a challenge in cropping and pasture situations. Understanding the factors that influence seed dormancy and germination will enable landholders to better predict the occurrence of *Pimelea* germination events in pastures and take proactive measures to avoid unnecessary adverse impacts on livestock.

## Figures and Tables

**Table 1:** Germination of *P. trichostachya* taken from pooled data from two experiments of three replicates of 50 single seeded fruits, either with GA_3_ applied to intact, mildly scarified or heavily scarified fruits.

Treatments *	Germination (%) in GA_3_ (mM)			
	0.00	0.58	1.15	1.73
Intact seed	0.0	0.0	0.0	0.0
Mild scarification	0.0	0.0	0.0	0.0
Heavy scarification	0.0	62 ± 1 b	86 ± 3 a	67 ± 1 b

* Mild scarification involved removal of exocarp alone whereas heavy scarification involved removal of exocarp, mesocarp and weakening of endocarp by sandpaper. Values indicate means ± SEM. Similar letters after data indicate insignificance (*p* > 0.05).

## Data Availability

Not applicable.
